# How common is fatty infiltration of the teres minor in patients with shoulder pain? A review of 7,367 consecutive MRI scans

**DOI:** 10.1186/s40634-021-00325-2

**Published:** 2021-01-29

**Authors:** William R. Aibinder, Derrick A. Doolittle, Doris E. Wenger, Joaquin Sanchez-Sotelo

**Affiliations:** 1 Department of Orthopaedic Surgery and Rehabilitation Medicine, SUNY Downstate Health Sciences University, Brooklyn, NY USA; 2 Department of Radiology, Mayo Clinic, Rochester, MN USA; 3 Department of Orthopedic Surgery, Mayo Clinic, 200 First Street SW, Rochester, MN USA

**Keywords:** Teres minor atrophy, Quadrilateral space, Shoulder pain, Magnetic resonance imaging

## Abstract

**Purpose:**

The teres minor is particularly important for activities that require external rotation in abduction in the settings of both rotator cuff tears and reverse shoulder arthroplasty. This study sought to assess the incidence of teres minor fatty infiltration in a large cohort of consecutive patients evaluated with shoulder MRI for shoulder pain and to identify all associated pathologies in an effort to determine the various potential etiologies of teres minor involvement.

**Methods:**

A retrospective review of 7,376 non-contrast shoulder MRI studies performed between 2010 and 2015 were specifically evaluated for teres minor fatty infiltration. Studies were reviewed by two fellowship trained musculoskeletal radiologists. Muscle atrophy was graded on a 3-point scale according to Fuchs and Gerber. The remaining rotator cuff tendons and muscles, biceps tendon, labrum, and joint surfaces were assessed on MRI as well.

**Results:**

In this series, 209 (2.8%) shoulders were noted to have fatty infiltration of the teres minor. The rate of isolated fatty infiltration of the teres minor was 0.4%. Concomitant deltoid muscle atrophy was common, and occurred in 68% of the shoulders with fatty infiltration of the teres minor. Tearing of the teres minor tendon was extremely rare.

**Conclusion:**

Fatty infiltration of the teres minor can occur in isolation, be associated with deltoid muscle atrophy only, or occur in the setting of rotator cuff full tears. Thus, fatty infiltration of the teres minor may be related to a neurologic process or disuse. Further long term longitudinal studies are necessary to be elucidate the etiologies.

**Level of Evidence:**

Level IV.

## Background

The importance of the teres minor has been emphasized recently in the settings of both rotator cuff tears and reverse total shoulder arthroplasty (RTSA) [2, 6, 22]. Several studies have demonstrated the teres minor muscle to be of utmost important for certain activities of daily living, in particular those that involve external rotation in abduction [1, 15, 20]. Nonetheless, it is the least commonly studied muscle of the rotator cuff [24]. Pathologic processes involving the teres minor in isolation are rare, but may include tearing of the musculotendinous insertion or neurologic denervation [9, 14, 18]. Tearing of the teres minor is uncommon, with reported rates as low as 0.9% of all rotator cuff tears [18]. Fatty infiltration of the teres minor muscle, however, has been reported to be more common in the setting of tears of other cuff tendons, with rates of approximately 3% [18, 21]. Furthermore, fatty infiltration of the teres minor has been shown to lead to worse outcomes following RTSA, latissimus dorsi tendon transfer, and treatment of rotator cuff tears [2, 6, 20, 22].

Sofka et al. reviewed 2,563 shoulder magnetic resonance imaging (MRI) examinations and reported isolated teres minor atrophy in 65 shoulders (2.5%) [21]. Melis et al. reviewed 1,572 shoulders with known rotator cuff tears on computed tomography (CT) or MRI studies, and noted teres minor involvement in 50 shoulders (3.2%) [18]. These studies provided some data regarding the rate of teres minor pathology in isolation and in association with rotator cuff tears, respectively. In many individuals, the etiology of fatty infiltration of the teres minor remains unclear, and it can be related to a quadrilateral space syndrome, a traction injury on the axillary nerve during trauma, chronic rotator cuff tearing or disuse from functional impairment due to associated injuries.

The purpose of this study was to assess the incidence of teres minor fatty infiltration in a large cohort of consecutive patients evaluated with shoulder MRI for shoulder pain and to identify all associated pathologies in an effort to determine the various potential etiologies of teres minor involvement.

## Methods

Following Institutional Review Board approval, a retrospective review of 7,376 consecutive non-contrast shoulder MRI studies performed between 2010 and 2015 for shoulder pain at our Institution were reviewed through a keyword database search including the terms: “teres minor,” “fatty infiltration,” “atrophy,” and “fatty degeneration.” MRI studies indicating teres minor abnormalities were then reviewed independently by two fellowship trained musculoskeletal radiologists (DAD, DEW) to grade fatty infiltration of the teres minor and identify any MRI evidence of other associated pathology.

Fatty infiltration of the teres minor was graded based on the 3-point classification system described by Fuchs et al. [10]. A grade of 0 represented no atrophy or a few fatty streaks, a grade of 1 represented less than 50% fatty infiltration (“more muscle than fat”) and a grade of 2 represented greater than 50% of fatty infiltration (“more fat than muscle”). Fatty infiltration was assessed on the axial and sagittal sequences. Specifically, the T1-weighted sagittal image at the level of the scapular spine and body forming a “Y”, was used to assess fatty infiltration of the 4 rotator cuff muscles. MRI was obtained on a 3 T MRI scan and protocoled based on the primary radiologist assigned to the case. Our radiologists graded not only the teres minor, but also the supraspinatus, infraspinatus, subscapularis, and deltoid muscles. Images were also assessed for relevant pathologic findings of the rotator cuff tendons, biceps tendon, labrum, as well as articular cartilage degenerative changes.

Means and ranges are reported for continuous data. No statistical analysis was performed as the data represented is purely descriptive.

## Results

### Teres Minor Abnormalities

**Fig. 1:**
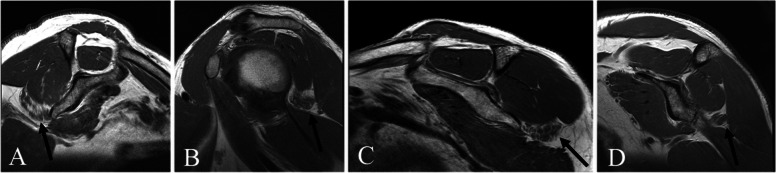
Sagittal T1-weighted MRI demonstrating fatty infiltration of the teres minor muscle in 4 patients

Teres minor abnormalities were noted in 209 (2.8%) shoulders, which comprised our study cohort. The mean age in the cohort was 60.6 years (range, 22 to 87 years) with 78% males. Fatty infiltration of the teres minor muscle was grade 1 in 91 (43%) shoulders and grade 2 in 118 (57%) shoulders (Fig. 1). In this cohort, no full thickness tears of the teres minor tendon could be identified. Two (1%) shoulders were noted to have partial thickness tearing of the teres minor tendon.

### Fatty Infiltration of the Remaining Cuff and Deltoid

**Fig. 2:**
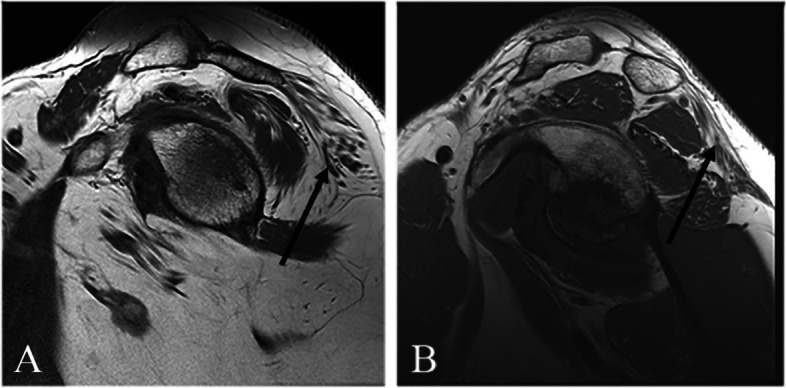
Sagittal T1-weighted MRI demonstrating fatty infiltration of the deltoid muscle in 2 patients

Fatty infiltration of the teres minor occurred in isolation in 27 shoulders (13%). Table 1 summarizes rates of fatty infiltration of the remaining rotator cuff muscles and the deltoid muscle. Concomitant deltoid muscle fatty infiltration was the most common, and found to occur in 143 shoulders (68%), with 95% of those being grade 1 and 5% being grade 2 (Fig. 2). Teres minor and deltoid fatty infiltration in combination, without any involvement of the other rotator cuff muscles, occurred in 59 shoulders (28%).

**Table 1:** Fatty infiltration of the rotator cuff and deltoid muscles based on the Fuchs-Gerber classification

	**Grade 0**	**Grade 1**	**Grade 2**
Teres Minor	0 (0%)	91 (43.5%)	118 (56.5%)
Supraspinatus	87 (41.6%)	64 (30.6%)	58 (27.8%)
Infraspinatus	81 (38.8%)	79 (38.8%)	49 (23.4%)
Subscapularis	105 (50.2%)	75 (35.9%)	29 (13.9%)
Deltoid	66 (31.6%)	135 (64.6%)	8 (3.8%)

### Rotator Cuff Tendon Tears

A full thickness rotator cuff tendon tear of the supraspinatus, infraspinatus, and subscapularis was identified in 100 shoulders (48%), 51 shoulders (24%), and 31 shoulders (15%), respectively (Table 2). Most tears of the subscapularis tendon did not involve the entire footprint from proximal to distal subscapularis.

**Table 2:** Rotator cuff tendon pathology associated with teres minor fatty infiltration

	**Normal**	**Tendinopathy**	**Partial Thickness Tear**	**Full Thickness Tear**
Teres Minor	203 (97%)	4 (2%)	2 (1%)	0 (0%)
Supraspinatus	1 (0.5%)	40 (19%)	68 (33%)	100 (48%)
Infraspinatus	4 (2%)	84 (40%)	70 (33%)	51 (25%)
Subscapularis	8 (4%)	73 (35%)	97 (46%)	31 (15%)

### Other Findings

The most common abnormal associated imaging finding involved the tendon of the long head of the biceps (207 shoulders, 99%). These findings included biceps tendinopathy in 60 shoulders (29%), partial tearing in 100 shoulders (48%), and full thickness tearing in 47 shoulders (22%). The labrum was noted to be abnormal in 192 shoulders (92%), with degenerative changes being the most common abnormality. Moderate or severe cartilage wear was noted in 65 shoulders (31%), with mild changes in an additional 69 (33%) shoulders.

## Discussion

The teres minor muscle serves an important biomechanical function as part of the posterior compressive forces required to maintain an adequate transverse force couple [4, 15]. In addition, the teres minor has been demonstrated in the literature to be important for external rotation, in particular with the arm abducted 90 degrees, with a direct impact on certain activities of daily living [1]. However, there is limited information in the published literature specifically investigating abnormalities of the teres minor.

The results of our study seem to indicate that, when considering the universe of individuals presenting with shoulder pain of any origin investigated with MRI, fatty infiltration of the teres minor can be expected in approximately 3% of the shoulders, and it is isolated in less than 1%. Fatty infiltration of the teres minor was commonly associated with fatty infiltration of the deltoid and the remaining muscles of the rotator cuff. However, tears of the teres minor tendon seem to be exceedingly rare.

A few other studies published in the peer reviewed literature have reported on teres minor abnormalities. Melis et al. reported a 0.9% rate of teres minor tearing, which was always associated with a supraspinatus and infraspinatus tear [18]. Of note, in their series, the cohort included only patients with rotator cuff tears. The same authors reported a 3.2% rate of atrophy of the teres minor [18]. The rate in our study was 2.8% with all shoulder MRI exams performed over a 6 year period as the denominator. This differed from the cohort of Melis et al., as their denominator only included patients with rotator cuff tears. The comparable rates in these two studies may be due to the fact that teres minor atrophy could be a consequence not only of direct tendon tearing, but also to kinking or compression of the axillary nerve or branches to the teres minor in patients with retracted posterosuperior cuff tears.

Sofka et al. reviewed all shoulder MRI exams performed at a single Institution during a two-year period and reported a 2.5% rate of isolated teres minor denervation based on the presence of fatty infiltration [21]. Cothran and Helms demonstrated a 0.8% rate of focal teres minor atrophy [7]. Kang et al. reported a 6.2% rate of isolated teres minor atrophy, of which only 41% was complete. The authors described the possibility of a partial type of fatty atrophy with two separate patterns [11]. In our series, isolated fatty infiltration of the teres minor only occurred in 27 shoulders (0.4%). Although the rate of isolated fatty infiltration is lower in our cohort, these studies demonstrate that isolated abnormalities of the teres minor is a relative rare, but real phenomenon. Kruse et al. reported on the only series of patients surgically treated for idiopathic isolated teres minor atrophy by performing decompression of the nerve to teres minor, with improvement in external rotation strength [14]. These patients presented with posterior shoulder pain and weakness with shoulder external rotation.

Quadrilateral space syndrome is a distinct entity first described by Cahill and Palmer [5]. In addition to the symptoms consistent with idiopathic isolated teres minor dysfunction, these patients will often have associated weakness of the posterior deltoid muscle and numbness in the axillary nerve distribution. This syndrome is secondary to compression of the axillary nerve and posterior circumflex humeral artery (PCHA), as these structures pass through the quadrilateral space [17]. True quadrilateral space syndrome is an extremely rare diagnosis; its true epidemiology is difficult to determine, as most diagnoses tend to be incorrect [3, 8]. Successful surgical treatment has been reported. Procedures are aimed at releasing fibrous bands, removing space occupying lesions, or treating vascular occlusion of the PCHA [5, 8, 16, 17, 19]. Based on the concomitant findings of isolated teres minor and deltoid atrophy, it is likely that subclinical imaging findings consistent with axillary nerve involvement are more common than florid symptomatic quadrilateral space syndrome. It is important to recognize that this subclinical imaging presentation may be prognostic of postoperative outcomes for various procedures.

Teres minor fatty infiltration has been reported to lead to worse outcomes after shoulder procedures, especially RTSA and rotator cuff repair. Simovitch et al. reported on 42 patients undergoing RTSA and found worse results in those with Goutallier stage 3 and 4 preoperative fatty infiltration of the teres minor [20]. The authors noted worse Constant scores, shoulder subjective values, and a net loss in external rotation. This study did not mention the status of the deltoid muscle on imaging. In contrast, Wiater et al. recently reported on 30 patients undergoing RTSA who were assessed with an MRI preoperatively [23]. With the numbers available, these authors could not detect an effect on outcomes based on the amount of teres minor fatty infiltration. They did, however, report worse postoperative ASES scores in patients with fatty infiltration of the deltoid muscle. It is possible that perhaps the worse results associated with RTSA in reports speculating teres minor weakness to be the culprit, are due to a combination of deltoid denervation and teres minor denervation. Posterior deltoid dysfunction could contribute to weakness in external rotation. Kim et al. demonstrated that fatty infiltration of the teres minor in patients undergoing a rotator cuff repair did not affect the functional outcomes [13].

The purpose of this study was to describe the imaging findings associated with teres minor fatty infiltration and atrophy. As such, our study has several limitations. First, detailed clinical information for shoulders included in this cohort were not analyzed. Many of the imaging studies were not requested by orthopedic surgeons, but rather by primary care providers in the community. Thus, a comprehensive musculoskeletal examination of the shoulder with dedicated teres minor maneuvers is not available for the majority of the patients included in this study. Furthermore, isolated teres minor dysfunction is a rare clinical presentation, and even if electromyography (EMG) and nerve conduction studies are ordered, the technician would need to be directed to specifically assess the teres minor muscle, uncommonly included in routine EMG testing. Second, the shoulders in this study were captured by initially performing a keyword search for the terms “teres minor,” “atrophy,” and “fatty infiltration” in a dedicated radiology database. Although, all flagged images were reviewed by two fellowship trained musculoskeletal radiologists, the overall incidence of teres minor abnormalities reported here may in fact be an underestimate. Third, this study only assessed fatty infiltration of the teres minor. Previous reports have also assessed teres minor hypertrophy. The latter has been shown to occur with both anterosuperior and posterosuperior rotator cuff tears [12, 18]. Thus, this study may not be capturing the entire constellation of teres minor findings.

This study does have several strengths. To our knowledge, this is the largest series of patients with teres minor abnormalities assessed on imaging. Most prior studies sought to identify the incidence of teres minor pathology within a particular subset of diagnoses and to determine the effects on outcomes [6, 18, 20, 21]. This study, however, sought to evaluate the pathologies associated with teres minor abnormalities.

As such, both orthopedic surgeons and radiologists alike should be astute to the teres minor muscle when reviewing an MRI or CT scan. It is often forgotten and overlooked, since the tendon of the teres minor is seldom torn compared to the rest of the rotator cuff. Furthermore, on physical examination, the teres minor muscle may be challenging to isolate, particularly in patients with an intact infraspinatus. Fatty infiltration and atrophic changes of the teres minor muscle may be seen as part of idiopathic isolated teres minor dysfunction, symptomatic or subclinical quadrilateral space syndrome, and rotator cuff disease. The previously reported poor outcomes for cuff repair, reverse shoulder arthroplasty and tendon transfers in the setting of teres minor atrophy could be partly explained by the high rate of concomitant deltoid muscle atrophy. Further clinical studies to assess the quality of the deltoid muscle specifically in patients with teres minor dysfunction are necessary.

## Conclusion

Fatty infiltration of the teres minor can be expected in approximately 3% of all patients undergoing MRI for the evaluation of a painful shoulder. There is variability in the concomitant findings that are seen associated with teres minor fatty infiltration. Based on the available data, it is unclear as to the exact etiology of fatty infiltration of the teres minor.
